# The role of antioxidant nutrients in mitigating PM_2.5_-related health risks in young Indian children

**DOI:** 10.3389/fpubh.2025.1575950

**Published:** 2025-05-09

**Authors:** Franciosalgeo George, Ekta Chaudhary, Sagnik Dey, Tinku Thomas, Harshpal Singh Sachdev, Anura Kurpad, Santu Ghosh

**Affiliations:** ^1^Centre for Doctoral Studies, Manipal Academy of Higher Education, Manipal, India; ^2^Division of Epidemiology, Biostatistics, and Population Health, St. John’s Research Institute, Bengaluru, India; ^3^Department of Epidemiology, University of Michigan School of Public Health, Ann Arbor, MI, United States; ^4^Centre for Atmospheric Sciences, Indian Institute of Technology Delhi, New Delhi, India; ^5^Faculty of Adjunct, Department of Health, Policy and Management, Korea University, Seoul, Republic of Korea; ^6^Department of Biostatistics, St. John’s Medical College, Bengaluru, India; ^7^Department of Pediatrics and Clinical Epidemiology, Sitaram Bhartia Institute of Science and Research, New Delhi, India; ^8^Department of Physiology, St. John’s Medical College, Bengaluru, India

**Keywords:** PM_2.5_ exposure, acute respiratory illness (ARI), anaemia, antioxidant nutrient intake, air pollution, child health

## Abstract

**Introduction:**

Pollution (PM_2.5_) exposure can result in acute respiratory illness (ARI) and anaemia in children. We aimed to investigate if antioxidant nutrient intakes could mitigate the impact of PM_2.5_ on child health outcomes on a national scale in India.

**Methods:**

We triangulated satellite-derived PM_2.5_ exposure data at the primary sampling unit level, with ARI and anaemia prevalence data from national district-level survey, and antioxidant nutrient intakes from household food expenditure survey. Logistic mixed effects regression model was used to estimate the effect of PM_2.5_ at different levels of nutrient intake.

**Results:**

This study included 208,782 children with valid ARI and 197,289 children with valid hemoglobin measurements. The prevalence of ARI and anaemia were 2.8% (95% CI: 2.3, 3.2) and 57.6% (95% CI: 57.2, 57.9) respectively. The intake of selected antioxidant nutrients such as vitamin C, D, and selenium, when higher than their estimated average requirement (EAR), lowered the risk of ARI associated with high PM_2.5_ exposure, while intakes higher than the EAR of vitamins A, C, D, zinc, and selenium similarly lowered the risk of anaemia. In terms of foods, similar benefits were observed with daily consumption of small amounts of fruits and vegetables.

**Conclusion:**

The result of this study highlights the importance of antioxidant rich balance diet for neutralizing adverse health effects of air pollution exposure to some extent till the environmental policy of the country could reduce emission of hazardous pollutants below safe level for human health.

## 1 Introduction

Exposure to air pollution poses significant health risks, particularly to infants and young children. For example, air pollution contributed to 31% of mortality in infants under 28 days old, and was responsible for 30% of fatalities attributed to lower respiratory infections (1, 2). In 2021, air pollution ranked as the second leading global risk factor for premature mortality, surpassed only by high blood pressure. Among children under five, it also ranked second as a cause of death, falling behind malnutrition (1). Remarkably, within the spectrum of environmental and occupational risks, air pollution emerges as the primary contributor to adverse health impacts, globally (3). India stands at the 8th position among the world’s most polluted countries, registering an annual average PM_2.5_ concentration of 53.3 μg/m^3^ in 2022, and over 10 times higher than the WHO PM_2.5_ guideline (4). The 2017 India state-level global burden of disease study estimated that 0.67 (UI: 0.55–0.79) million deaths in India, constituting 12.5% of total deaths, were linked to ambient PM_2.5_ (5).

Acute respiratory infections (ARI) and anaemia are major contributors to childhood morbidity and mortality globally, particularly in low and middle-income countries (6). Children are highly susceptible to these conditions due to their developing immune systems and higher metabolic needs. A growing body of evidence highlights ambient and household air pollution, particularly fine particulate matter (PM_2.5_), as a critical environmental risk factor for both ARI and anaemia in children (7, 8). According to the 2023 World Health Statistics, ARI stands as the leading cause of childhood mortality among all infectious diseases (9). Exposure to PM_2.5_ has also been shown to increase the risk of anaemia among children (6, 10).

PM_2.5_, which consists of aerodynamic particles with diameter less than 2.5 microns, penetrates deep into the respiratory tract and reaches the alveoli, causing inflammation, oxidative stress, and impaired lung function (11). Epidemiological studies have consistently shown that PM_2.5_ exposure increases the risk of lower respiratory tract infections such as pneumonia and bronchitis in children (12, 13). Moreover, chronic exposure to PM_2.5_ has been implicated in systemic inflammation and dysregulation of iron metabolism, contributing to anaemia (14).

Recent research is increasingly exploring nutritional interventions as potential modifiers of PM_2.5_^−^ related health risks. Antioxidant-rich micronutrients such as vitamins A, C, E, and trace elements like zinc, selenium, and iron have been shown to reduce oxidative stress and inflammation—key mechanisms underlying air pollution–related health outcomes (15). For example, vitamin C and E have protective roles in lung function through their reactive oxygen species (ROS)-scavenging properties, while iron and zinc are essential for erythropoiesis and immune function, which can be compromised in polluted environments (16). In children exposed to biomass smoke or high ambient PM_2.5_, iron deficiency anaemia may be worsened by inflammation-induced hepcidin expression, leading to impaired iron absorption and recycling (17). Nutritional supplementation, particularly with iron and antioxidants, has shown promise in reducing the burden of both ARI and anaemia in pollution-exposed populations (18, 19). However, there is limited evidence on whether antioxidant nutrients can modify the impact of PM_2.5_ on these outcomes in children. Most existing studies focus on adult populations (20, 21).

To our knowledge there is no comprehensive study that has explored, on a national scale, the mitigating influence of antioxidant nutrient intake on the relationship between PM_2.5_ exposure and child health outcomes, nor has there been any attempt to define specific nutrient or food intake requirements for this situation. The present study used satellite-derived PM_2.5_ exposure, along with triangulated data from two national district-level surveys with high granularity (see Methods), that captured nutrient intake and child health, to evaluate if specific nutrient and/or food intakes could mitigate the impact of PM_2.5_ exposure on specific child health outcomes, and if so, to define the additional daily requirements of these nutrients or foods in this specific functional domain.

## 2 Materials and methods

### 2.1 Data and sample

We utilized two nationally representative and granular Indian datasets for this study. The first dataset was the National Family Health Survey-4 (NFHS-4) conducted during 2015–2016, which provided information on acute respiratory infections (ARI) in children aged 6–59 months, as well as data on household sociodemographic and maternal characteristics. The second was the 68th round survey (2011–2012) of the National Sample Survey Office (NSSO), which provided information on household food and nutrient consumption.

The NFHS-4 was a nationally representative survey conducted between January 2015 and December 2016, covering both urban and rural areas at the district level, across 29 states and 6 Union Territories of India. A total of 221,858 children aged 6–59 months from 156,038 households were included in the survey. The survey achieved a high response rate of 97.6% for households and 96.7% for eligible women. Information on socioeconomic status, reproductive health and family planning, maternal and child health, and the occurrence of ARI-related symptoms were obtained from these children and households (22).

The ninth quinquennial Household Consumer Expenditure survey of the NSSO, known as NSS 68, was conducted in the same 29 states and 6 Union Territories, and included 59,683 rural households and 41,968 urban households. This survey collected data on the monthly per capita consumer expenditure, along with household food purchases of 223 food items, for a 30-day recall period (23). The quantities of food purchased were converted into nutrients using the Indian food composition table, with food items listed by number, or cost-converted into food weights (24). The per capita daily nutrient intake was calculated by dividing the total daily nutrient purchased by the household size. The rationale behind choosing NFHS-4 was to capture health data closer to NSSO 68th round and already validated mapping of NSSO 68th round on NFHS-4 (25).

### 2.2 Ambient PM_2.5_ exposure

We used the satellite-derived ambient PM_2.5_ concentration at the primary sampling unit (PSU) level as the air pollution exposure for all children residing in that PSU as identified by geocodes reported by NFHS-4 (22). PSUs refer to villages in rural regions and Census Enumeration Blocks (CEBs) in urban settings. These were identified using data from the 2011 Census, and any PSU with fewer than 40 households was merged with the nearest PSU to maintain sufficient sample sizes (22). Monthly average PM_2.5_ concentrations at PSU level were used to construct long term exposure due to lack of resolution at daily scale during the study period. The daily reported level-2 aerosol optical depth (AOD) from the Moderate Resolution Imaging Spectroradiometer (MODIS) at a 1 × 1 km^2^ spatial resolution was converted to daily surface PM_2.5_ using a dynamic scaling factor from Modern-Era Retrospective analysis for Research and Applications Version 2 (MERRA-2) reanalysis data. The instantaneous PM_2.5_ was then converted to a 24 h average using the diurnal scaling factor from MERRA-2. These scaling factors were calibrated against existing ground-based measurement by network of the Central Pollution Control Board of India (CPCB). The satellite-derived PM_2.5_ showed a high correlation (r^2^ = 0·97) and a root mean square error of 7·2 μg/m^3^ with values reported from coincident monitoring sites (26). For determining the risk for ARI and anaemia, we calculated the average early-life exposure from birth to interview month, to capture the life-course PM_2.5_ exposure of children who were 6–59 months old at the time of assessment. For example, if a child was born on January 2014 and the NFHS-4 interview took place on December 2015, we computed the average of the daily PM_2.5_ values from January 2014 through December 2015 for the PSU where the child resided.

### 2.3 ARI and anaemia outcomes

ARI was identified in children aged 6–59 months old based on maternal reports in the NFHS-4 Children’s Recode (KR) file. Specifically, children were considered to have ARI if they had symptoms such as coughing, along with quick or troubled breathing in the 2 weeks before the survey. Anaemia status was also obtained from the KR file. The presence of anaemia in these children was diagnosed from the hemoglobin (Hb) concentration in capillary blood samples collected by the finger or heel prick technique, which was measured immediately on site using the HemoCue Hb 201 + analyzer (27). Children were classified as anaemic if their Hb concentrations were below 11 g/dL (28). Both ARI and anaemia was treated as a binary variable (yes/no) for the analysis.

### 2.4 Data triangulation

The household-level data from the NFHS-4 and NSSO surveys were combined for the same district in a state using triangulation. Here, the NSSO-68 data on food and nutrient intake served as the donor dataset, while the NFHS-4 household survey data was the recipient. To match the datasets, a set of common variables, including family size, religion, locality (rural/urban), and socioeconomic status, which were available in both NFHS4 and NSS68 surveys were selected, and the nearest-neighbour hot deck method was employed for triangulation. This triangulation method has been validated earlier (25).

### 2.5 Selection of nutrients

The micronutrients were selected based on their anti-oxidant property and scientific evidence for protective effects on incidence of ARI and anaemia along with existing mitigation effects on adverse health effects of air pollution in the literature. According to above criteria we chose vitamin D, selenium, vitamin B_12_, vitamin C, zinc and vitamin A (26–29). The per capita intakes of these nutrients were extracted from the triangulated data set, as described above, for analysis of their potential effect mitigation on the risk of ARI and anaemia related to PM_2.5_ exposure.

### 2.6 Confounding covariates

The analyses included a set of potential individual-level and household-level covariates. The individual-level variables included the child’s sex (boy or girl) and the mother’s education level (no education, primary, secondary, or higher). The household-level covariates included socioeconomic status, which was categorized into five wealth quintiles (poorest, poor, middle, rich, and richest), the type of residence (rural or urban), the type of cooking fuel (categorized as: (1) clean fuels – including electricity, liquefied petroleum gas (LPG)/natural gas, and biogas; (2) solid fuels – including coal/lignite, charcoal, wood, crop residues, dung cakes, and other biomass; and (3) kerosene), Frequency of smoking within household and household size.

### 2.7 Statistical analyses

We assigned the PSU-level average (over time) ambient PM_2.5_ concentration as the air pollution exposure for the children who lived in the PSU. We applied a logistic mixed effects model to account for cluster effects at PSU. We emphasised on modification of the association between child health and PM_2.5_ air pollution by per capita intake of six nutrients with anti-oxidant properties as listed above. The following regression model was used for analysis:


$$\begin{array}{l} \mathrm{logit}\left\{\mathrm{Prob}\left(Y_{ij}=1|u_{i}\right)\right\}=\beta_{0}+\beta_{1}{\left(PM_{2.5}\right)}_{ij}+\\ \beta_{2}\left[{\left(PM_{2.5}\right)}_{ij}\times {\left(\mathrm{Nutrient}\right)}_{ij}\right]+\\ \gamma X_{ij}+u_{i} \end{array}$$

where $Y_{ij}$ represented the binary outcome (ARI/anaemia) for the *j*^th^ individual in *i*^th^ PSU, ${\left(PM_{2.5}\right)}_{ij}$ was the average life course exposure of the child, ${\left(\mathrm{Nutrient}\right)}_{ij}$ was the per capita nutrient intake of household of *i*^th^ child of *j*^th^ PSU, u_i_ was a random intercept corresponding to *i*^th^ PSU with $u_{i}∼N\left(0,\sigma_{u}^{2}\right)$ , $\left(\beta_{0},\beta_{1},\beta_{2}\right)$ were intercept and slopes of interest, respectively, and γ represented regression coefficients corresponding to confounders adjusted in the respective model. $\beta_{1}$ was interpreted as the slope of PM_2.5_ at the lower selected nutrient intake while $\beta_{2}$ was interpreted as the change in slope of PM_2.5_ at the higher selected level of nutrient intake against the lower level intake. Mitigation of risk by the higher intake of nutrient was considered significant if the estimate of $\beta_{2}$ was negative and statistically significant (*p* < 0.05).

To define the “adequate intake” of selected nutrients that might mitigate the effects of PM_2.5_ on ARI and anaemia, the intake of these nutrients was categorized into intervals of <10th, 10–25th, 25–50th, 50–75th, 75–90th and >90th percentiles. The percentile of nutrient intake above which there was a decline in slope of the relation between the OR of anaemia or ARI and PM_2.5_ exposure was considered to be an “adequate” intake. The change in the slope of the relation between OR of anaemia or ARI and PM_2.5_ exposure as the nutrient intake further increased above the adequate intake was also examined. The nutrient intake levels retained for this analysis was based on the presence of an adequate (>10% change against the previous category, assumed to be adequate) and statistically significant change in slope as nutrient intake increased.

A similar exercise was carried out with the per capita fruit and vegetable intake (since these are sources of antioxidant micronutrients in the normal Indian diet (29)), to translate our findings with the nutrient associations with mitigation of PM_2.5_ effects, if any, into food-based recommendations. To visualize the risk of anaemia/ARI with increasing PM_2.5_ exposure at different levels micronutrient intake the above logistic regression model was used to predict the risk at selected level of micronutrient intakes. R version 4.3.3 ((55), Vienna, Austria) was used for all statistical analyses.

## 3 Results

Out of the original 221,858 observations on 6–59 month old children in the NFHS-4 dataset, the data of 208,782 children with valid ARI information and 197,289 children with valid Hb measurements were extracted. The baseline characteristics of the study participants included in the ARI and anaemia analyses are summarized in Table 1. The majority of children resided in rural areas (76.1%), with nearly 70% of households using unclean cooking fuel and over one-third reporting daily indoor smoking exposure. The estimated prevalence of ARI was 2.8% (95% CI: 2.3, 3.2) and 57.6% (95% CI: 57.2, 57.9) children were anaemic. The prevalence of ARI and anaemia across different levels of confounders is reported in Supplementary Figure 1. The mean life course exposure to PM_2.5_ for 6–59 month old children was estimated to be 67.7 ± 16.6 μg/m^3^ and the distribution varied from 20 to ~100 μg/m^3^. The unadjusted and adjusted logistic regression models estimated OR of ARI as 1.12 (95% CI: 1.10–1.14) and 1.17 (95% CI:1.16–1.18) respectively, for every 10 $\mathrm{\mu g}/m^{3}$ increase in ambient PM_2.5_ exposure. Similarly, the estimated OR of anaemia for every 10 $\mathrm{\mu g}/m^{3}$ increase in ambient PM_2.5_ exposure was estimated as 1.18 (95% CI: 1.17–1.18) for the unadjusted model and 1.14 (95% CI: 1.13–1.14) for the adjusted model. The confounders that were adjusted for included child age and sex, mother’s education, locality, wealth quintile, household size, type of cooking fuel, passive smoking indicator, along with cluster effects at PSU level. The per capita daily iron intake was also adjusted for in analyses of anaemia.

**Table 1:** Characteristics of the study population (children aged 6–59 months).

Variable	Frequency (%)^a^
Sex of child	
Male	102,774 (52.1%)
Female	94,515 (47.9%)
Education of mother	
Illiterate	61,651 (31.2%)
Primary	29,262 (14.8%)
Secondary	88,401 (44.8%)
Higher	17,975 (9.1%)
Wealth index	
Q1	52,249 (26.5%)
Q2	46,373 (23.5%)
Q3	39,247 (19.9%)
Q4	32,831 (16.6%)
Q5	26,589 (13.5%)
Urban/Rural	
Rural	150,069 (76.1%)
Urban	47,220 (23.9%)
Household size	6 (5.0, 8)^a^
Cooking fuel	
Clean fuel	57,993 (29.4%)
Kerosine	1,342 (0.7%)
Unclean fuel	137,954 (69.9%)
Frequency of smoking within household	
Never	96,083 (48.7%)
Less than monthly	7,042 (3.6%)
Monthly	6,997 (3.5%)
Weekly	18,789 (9.5%)
Daily	68,378 (34.7%)

a Median (Q1, Q3).

### 3.1 Risk reduction by dietary intake of select micronutrients

The geometric mean of the per capita daily dietary intake of vitamin D (D2, D3 and 15(OH)2D) was estimated at 23.2 μg. Similarly, the mean per capita daily dietary intake of selenium was estimated at 87.7 μg, vitamin B_12_ at 0.68 μg, vitamin C at 43.1 mg, zinc at 9.8 mg and vitamin A at 162.5 μg (Supplementary Figure 2). To avoid undue variation, the lower and upper 0.5% of the intake measurements were excluded.

**Figure 1:**
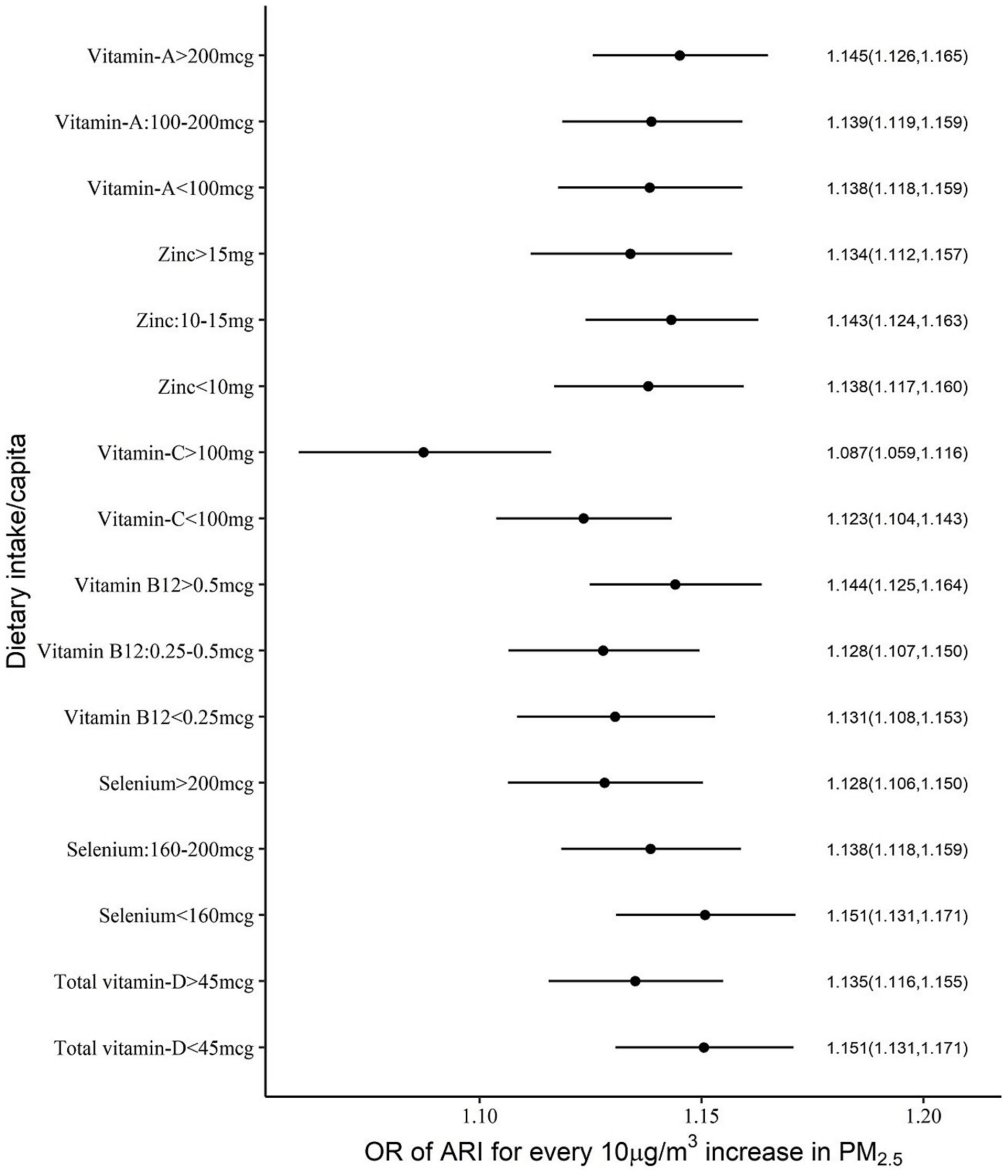
Adjusted OR (with 95% CI) of ARI among <5y Indian children over different levels of per capita daily intake of selected micronutrients with antioxidant properties.

**Figure 2:**
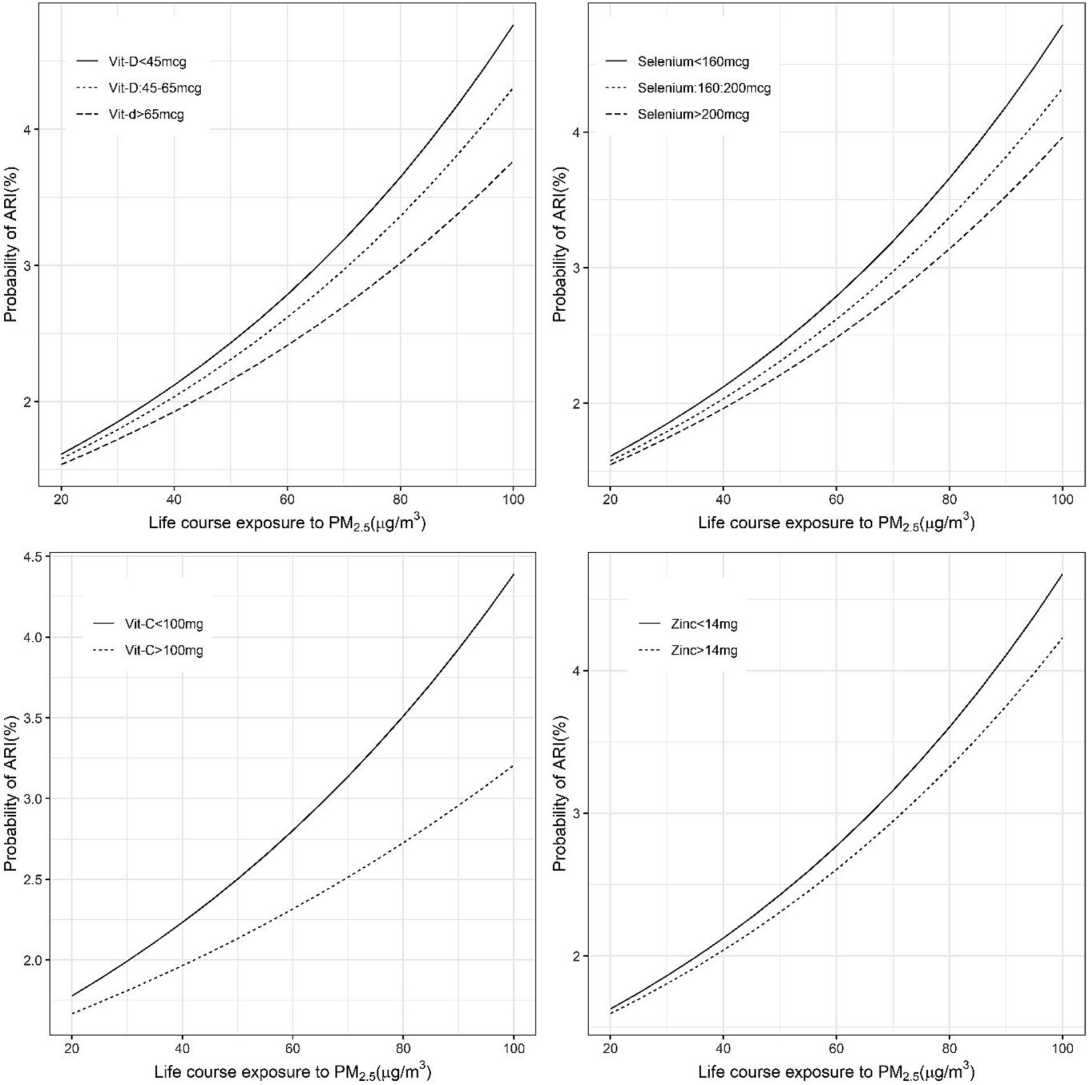
Exposure-response figure of the prevalence of ARI and ambient PM_2.5_ exposure for children with different household per capita intakes of antioxidant micronutrients.

Significant negative effects of nutrient intake in the OR for PM_2.5_ exposure on anaemia or ARI were considered as evidence of mitigation of risk by the selected nutrients. The different cut-off intake levels for <5y children, that were estimated for a functional effect on the PM2.5 exposure relationship, are provided in Table 2. The adjusted OR of ARI for every 10 μg/m^3^ increase in PM_2.5_ was mildly reduced by selenium, vitamin C and vitamin D intake, with a significant negative (meaning a mitigation effect) interaction. The estimated OR of ARI for every 10 μg/m^3^ increase in PM_2.5_ at different daily intakes of selenium, of <160 μg, 160–200 μg and >200 μg, showed a declining dose response of 1.15, 1.14 and 1.13, respectively. At a vitamin C intake of <100 mg/day the OR of ARI was estimated at 1.23, versus 1.08 when the intake was >100 mg/day and the estimated OR at a vitamin D intake >45 μg/day also was slightly lower (1.13 vs. 1.15) than at a daily intake <45 μg/day (Supplementary Table 1; Figures 1, 2).

**Table 2:** Cut off intakes for mitigation effects of nutrients calculated for <5y children and the corresponding estimated average requirements (EAR).

Nutrient intake	^a^Cut off intake	EAR
Vitamin D (μg/day)	22.5	10
Selenium (μg/day)	80	40
Vitamin B_12_ (μg/day)	0.125	1.5
Vitamin C (mg/day)	50	25.5
Zinc (mg/day)	5	3.25
Vitamin A (μg/day)	50	210

a Half of the per capita cut off assuming consumption unit of 0.5 for under 5y children.

**Figure 3:**
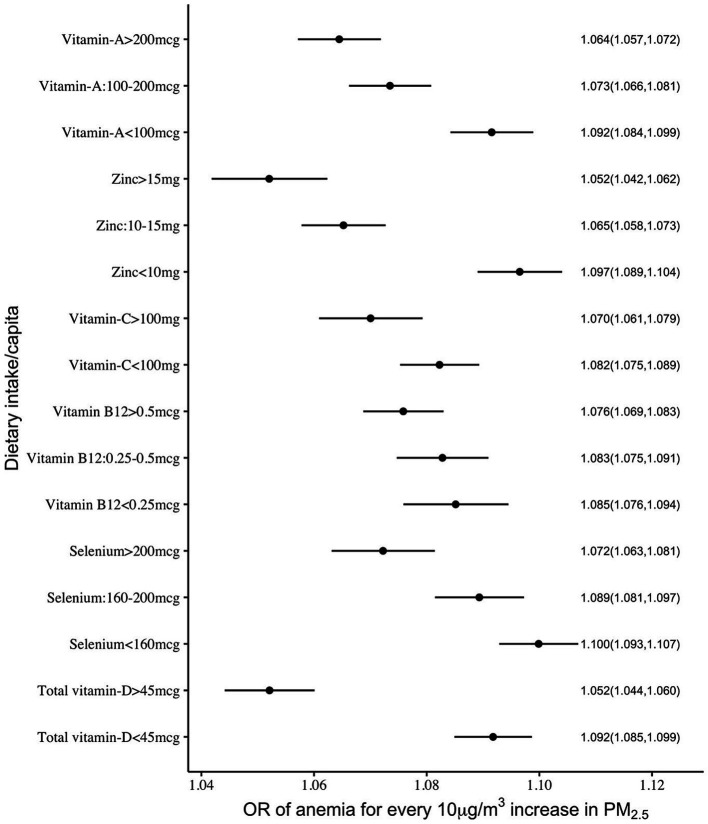
Adjusted OR (with 95% CI) of anaemia among <5y Indian children over different levels of per capita intake of selected micronutrients with antioxidant properties.

**Figure 4:**
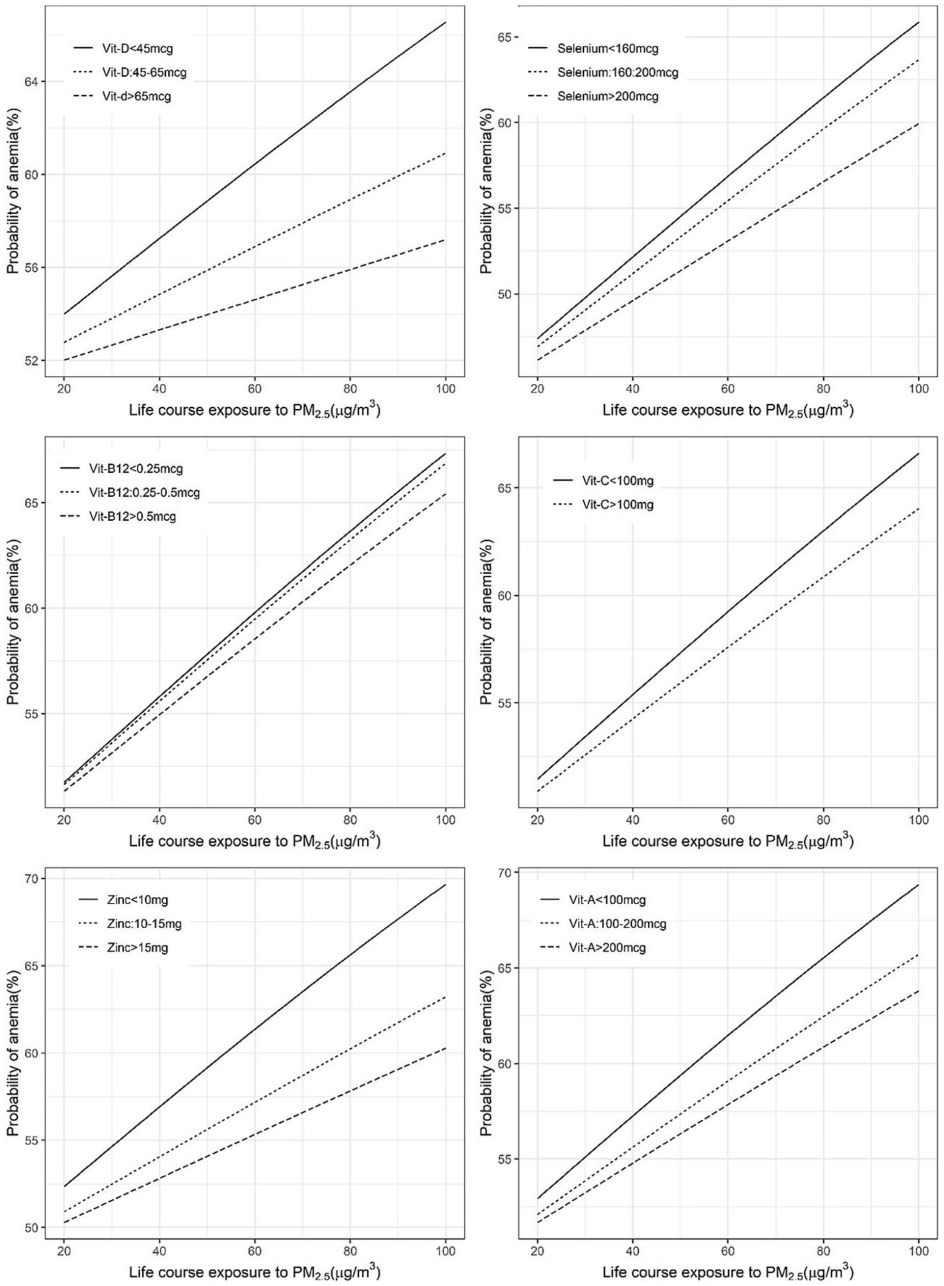
Exposure-response figure of the prevalence of anaemia and ambient PM_2.5_ exposure for children with different household per capita intakes of antioxidant micronutrients.

The mitigation by per capita daily nutrient intake was more uniform in the association between anaemia and PM_2.5_ exposure, where all selected nutrient intakes showed a significant negative interaction. The adjusted ORs of anaemia for every 10 μg/m^3^ increase in PM_2.5_ were estimated as 1.09 and 1.05 at daily per capita intakes of vitamin D of <45 μg and >45 μg, respectively. Similarly, at daily selenium intakes of <160 μg, 160–200 μg and >200 μg, the estimated ORs were 1.10, 1.09 and 1.07, respectively. The ORs at a daily vitamin C intake of <100 mg and >100 mg were estimated as 1.08 and 1.07, respectively. The estimated ORs at daily zinc intake levels of <10 mg, 10–15 mg and >15 mg were 1.10, 1.07 and 1.05, respectively. A similar pattern of OR estimates were observed (1.09, 1.07, 1.06) for different levels of daily vitamin A intake at <100 μg, 100–200 μg and >200 μg (Supplementary Table 2; Figures 3, 4).

**Figure 5:**
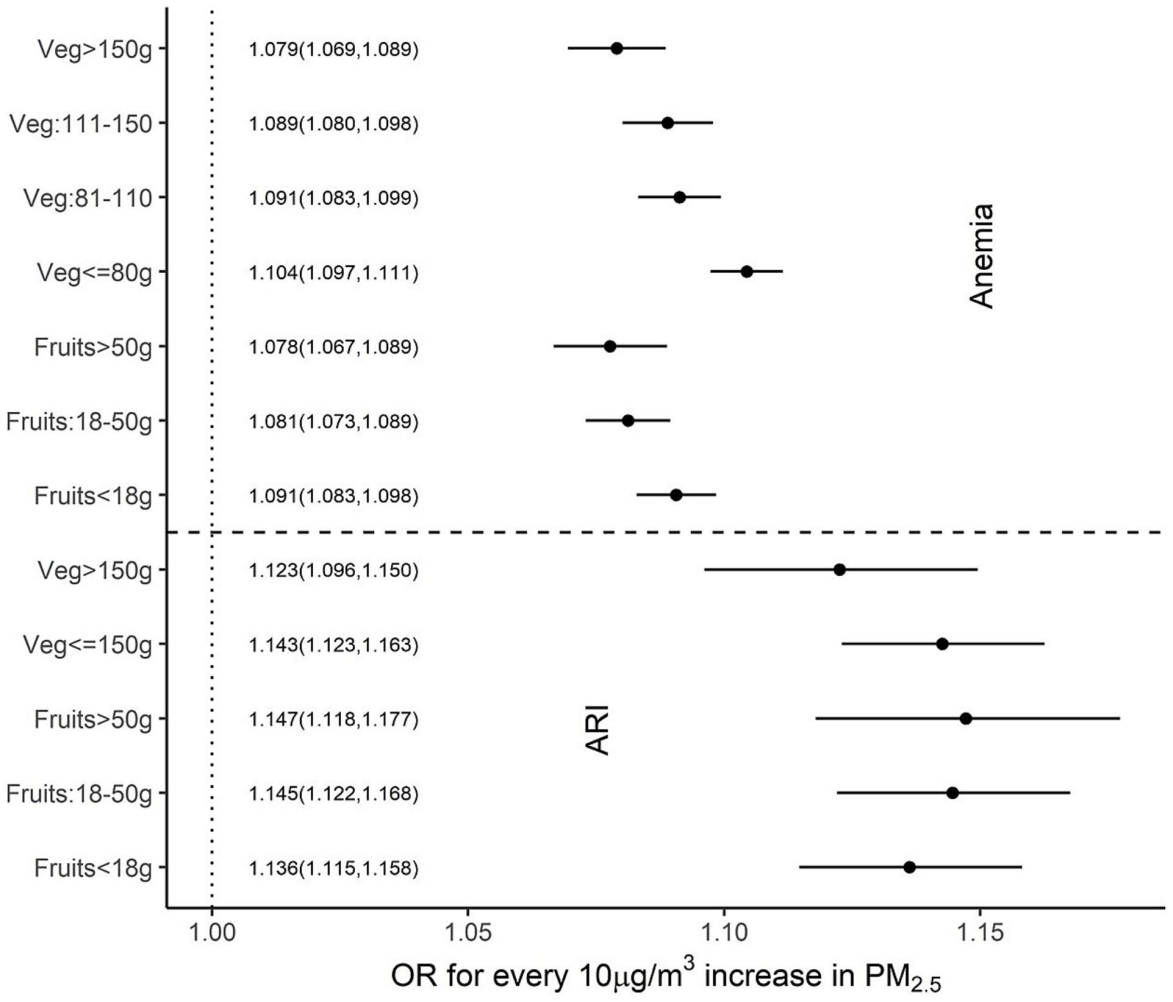
Adjusted OR (95% CI) of ARI and anaemia among children over different per capita fruit and vegetable intakes.

When similar associations were explored with per capita daily fruit and vegetable intake, higher intakes were associated with a reduced impact of PM_2.5_ on anaemia and ARI, but these were relatively weak compared to nutrient associations. A significant effect modification was observed with daily per capita vegetable intake of more than 150 g for ARI (OR 1.23 vs. 1.43) but not with fruit intake. For anaemia, a daily per capita fruit intake that was more than 18 g showed a significant effect modification (OR: 1.09, 1.08 and 1.08 for <18 g, 18–50 g and >50 g daily intake, respectively), while a daily per capita vegetable intake of more than 80 g had a significant reduction in the impact of PM_2.5_ on anaemia. The estimated ORs of anaemia, for every 10 μg/m^3^ increase in PM_2.5_, were 1.1, 1.09, 1.09 and 1.08 for daily per capita vegetable intakes of ≤ 80 g, 81–110 g, 110–150 g and >150 g, respectively (Figure 5).

## 4 Discussion

This study explored the potential mitigation of the effects of PM_2.5_ on child health outcomes such as ARI and anaemia by what we have termed an adequate intake of selected nutrients that had antioxidant properties. The normal required daily nutrient intake for a population is defined by its estimated average requirement (EAR). This is usually defined in biochemical terms as the average amount required to replace daily losses in a healthy population (30); typically, a functional or clinical approach to these requirements is not followed. The EAR of the selected nutrients is provided in Table 2. In comparison, for mitigation of adverse effects on ARI or anaemia, the adequate intake of each of these micronutrients, based on the statistical framework defined below, is also provided in Table 2. These statistical cut-offs of requirement were used to evaluate a functional benefit. Thus, an adequate daily per capita intake of vitamin C (>100 mg), vitamin D (>45 μg) and selenium (>160 μg), but no other micronutrient, showed a significant reduction in effects of PM_2.5_ on ARI prevalence (Figure 1). The highest benefit with these adequate nutrient intakes was observed in anaemia. Here, an adequate intake of all six selected micronutrients significantly reduced the effects of PM_2.5_ anaemia prevalence (Figure 3). Subsequently, adequate intake of fruits and vegetables, which are primary source of these micronutrients also demonstrated potential mitigation of the effects of PM_2.5_ on child health outcomes (Figure 5).

### 4.1 Mechanistic pathway

The link between antioxidant nutrients and inflammation is complex. Inflammation is at the heart of the adverse effects of PM_2.5_ exposure. Many studies that have shown a relation between PM_2.5_ exposure and risk of cardiac morbidity (31), altered respiratory responses to physical activity (32) and all cause mortality (33, 34), and the mechanism by which these associations occur, appear to be related to an inflammatory response with accompanying functional effects. For example, in a controlled human 4 h PM_2.5_ exposure (to a mean value of 37.8 μg/m^3^), a significant increase occurred in vascular inflammation and acute phase injury markers like serum amyloid A, C-reactive protein, soluble intercellular adhesion molecule-1, and soluble vascular cell adhesion molecule-1. Some of these remained elevated for almost a day after the acute PM_2.5_ exposure, and this was accompanied by a decreased pulmonary function. This is relevant to the aetiology of ARI. Systemic effects of inflammation include the suppression of intestinal iron absorption (35), which is an important erythropoietic nutrient, and can also suppress the erythropoietic activity of the bone marrow (36), leading to anaemia. It should be noted that these effects occurred with an acute exposure to a PM_2.5_ concentration that was not overly high, and close to the Indian national ambient air quality standard (NAAQS) value of 40 μg/m^3^ (37).

### 4.2 Role of select dietary micronutrients in mitigating impact of air pollution

The mitigating effects of antioxidants on the association between air pollution exposure and human health have been studied before (38–42). Antioxidant nutrients reduce inflammation by inducing the suppression of pro-inflammatory cytokines, affecting the expression of transcription factors involved in the immune response, and inhibiting key signalling pathways and enzymes involved in immune processes (43). For example, vitamin C, through its antioxidant properties, can neutralize free radicals through different mechanisms including the elimination of free radicals and reactive oxygen or nitrogen species, the down-regulation of enzymes producing free radicals, and the modulation of Nuclear Factor Erythroid 2-Related Factor and Nuclear Factor Kappa B (NF-κB), which are important mediators in oxidative stress (44). Selenium has antioxidant properties through selenoproteins that can protect against reactive oxygen species (45). Vitamin A and carotenoids can also be effective antioxidants. Their antioxidant activity is conferred by the hydrophobic chain of polyene units that can quench singlet oxygen, neutralize thiyl radicals and combine with and stabilize peroxyl radicals, being most effective at low physiological tissue oxygen tension levels (46). Zinc supplementation has been shown to increase plasma antioxidant capacity, decrease plasma inflammatory cytokines and oxidative stress biomarkers, as well as NF-κB activation (47). B vitamins and vitamin D can also mitigate the detrimental health effects of exposure to air pollution to some extent (48). Low levels of 25-hydroxy vitamin D enhanced adverse respiratory effects associated with indoor PM_2.5_ levels in obese urban children with asthma (49). Conversely, higher 25-hydroxy vitamin D levels had a protective role against asthma symptoms in homes with increased PM_2.5_, with estimated OR of 0.87. Vitamin B_12_ has potent antioxidant properties, including the direct scavenging of reactive oxygen species, preservation of glutathione levels, and the reduction of homocysteine-induced oxidative stress (50).

#### 4.2.1 Comparison with previous studies

Shin and Kim (20) found that higher intake of vitamin C and *β*-carotene attenuated the impact of long-term PM2.5 exposure on diabetes risk in Korean adults. Another study by Govindaraju et al. (21) showed that better overall diet quality moderated the long-term respiratory effects of PM2.5 exposure following the Hazelwood coalmine fire in Australia, further supporting the protective role of diet in pollution-related respiratory. In addition, Wang et al. (51) reported that adherence to a plant-based dietary pattern reduced the risk of COPD associated with long-term exposure to PM_2.5_, NO₂, and NO_x_. However, these studies primarily focus on adult populations. To our knowledge, no study has evaluated the nutrient-specific moderating effects on PM2.5-related risks in children using nationally representative data. Our study addresses this gap by identifying specific intake thresholds of antioxidant nutrients that may mitigate the risks of ARI and anaemia in Indian children under five.

### 4.3 Recommended dietary allowance under high air pollution exposure

The “adequate” nutrient intakes that were identified for a putative mitigating benefit on the risk of ARI or anaemia from PM_2.5_ exposure were almost double those of the EAR for vitamin D, C, selenium and zinc, but a fraction of the vitamin B_12_ and vitamin A EAR (Table 1). The EAR is usually based on the replacement of the daily nutrient loss in healthy individuals with no environmental stress, but frameworks can be defined for the prevention of specific morbidities or disease management. There are several challenges and uncertainties in this process (52), and key scientific challenges encountered in the use of chronic disease endpoints to establish nutrient requirement values have been reviewed (53). However, the specific framework of nutrient requirements for risk mitigation from environmental exposures, particularly in this instance, of conditions emanating from deficiencies, has not been explored.

It seems logical that the mitigating effect of antioxidant nutrients on risk may be observed at intakes that are higher than their EAR. However, the lower than EAR cutoff values for the mitigating effects of vitamin B_12_ and vitamin A indicate that even small intakes of some nutrients (within their “healthy” EAR) may be adequate. Of interest, these particular nutrients (vitamin B_12_ and preformed vitamin A) are found in animal foods. This analysis also identifies, for the first time, a possible approach to define the adequate requirement of nutrients under adverse environmental exposures. This is particularly relevant in countries like India where the prevalence of micronutrient deficiencies and air pollution are very high.

### 4.4 Balanced diet, a potential defender for adverse effects of air pollution

The other noteworthy finding was that all nutrients were protective for the risk of anaemia, indicating that the best way for mitigation would be a balanced, diverse diet with high-quality foods that deliver adequate nutrients for this purpose. Thus, single nutrient approaches are unlikely to be helpful, and food-based approaches may be best. When analysed by food groups however, these associations were much attenuated. This is not altogether surprising, as the major contributor to the fruit group is banana, and not coloured fruits that contain the most antioxidants, although some fruits are eaten generously during their season (54). Similarly for vegetables, the major food contributors are potato and onion. Tomato is also eaten in high quantities depending on the season and can offer a significant intake of antioxidants. Nevertheless, there was a mitigating effect of about 80–150 g of vegetables for the impact of PM_2.5_ on ARI and anaemia, while there was some modification of the impact on anaemia with 18 g of fruits or more. This is significant given the usual intake of vegetables and fruits in India, of 145 and 15 g, respectively, for rural India, and 155 and 29 g for urban India (54). However, when suggesting the increase of fruits and vegetables, even in small amounts, it is best to be more specific about those that might be beneficial through their antioxidant content.

A key strength of this study is the use of large, nationally representative datasets—NFHS-4 and NSSO-68—that provide information on child health, household sociodemographic characteristics, and food consumption patterns across diverse regions in India. This broad coverage enhances the generalizability of our findings and allows for nuanced analysis of environmental and dietary exposures. One of the limitations of the study was that daily per capita intake was estimated from the household expenditure on monthly food consumption, rather than by direct individual food intake recall. In addition, since the food intake of children varies by age, the per capita intake attributed to them might only be a crude indicator that may result in an inaccurate estimation of true modification effects. Additionally, the ARI outcome was based on maternal self-report of symptoms, which may introduce recall bias. Since we assigned the same PM_2.5_ exposure value to all children residing within a given PSU, there is potential for exposure misclassification due to within-PSU variability in actual exposure that could not be accounted for. We also assumed that the child’s place of residence remained the same from birth until the time of the survey when calculating PM_2.5_ exposure. To protect subject confidentiality, NFHS displaces geolocations by up to 2 km in urban and up to 5 km in rural areas, with 1% displaced up to 10 km, which may contribute to exposure misclassification. In addition, due to lack of adequate temporal resolution of satellite derived PM_2.5_ data at daily scale, the potential mitigation effects of micronutrient could not be examined for short term effects of PM_2.5_ exposure on child health.

## 5 Conclusion

The finding from this study should be treated as qualitative evidence of potential moderation of the air pollution and health association by antioxidants and antioxidant rich food groups. Using nationally representative data, we identified specific intake thresholds of micronutrients such as vitamin C, vitamin D, selenium, and others, that may reduce the risk of PM_2.5_-related ARI and anaemia in children under five. These results highlight the potential of dietary strategies in mitigating the adverse effects of air pollution. However, the evidence now needs validation by community-based invention studies or randomized control trials with select antioxidants. While long-term solutions must focus on reducing the root causes of air pollution, such structural changes may take time, especially in developing countries like India. In this context, food-based approaches—particularly increasing the intake of fruits and vegetables—may offer a feasible and complementary pathway to protect vulnerable populations.

## Data Availability

The demographic and nutrition datasets used for this research is publicly accessible. Datasets from the National Family Health Survey (NFHS) are available on the Demographic and Health Surveys (DHS) program's website (https://dhsprogram.com/data/Using-Datasets-for-Analysis.cfm), while the National Sample Survey (NSS) datasets can be accessed through the website of the Ministry of Statistics and Programme Implementation, Government of India (http://mospi.gov.in/national-sample-survey-nss). Exposure datasets are available upon request to corresponding author.
