# Supplementary material for: The role of antioxidant nutrients in mitigating PM2.5-related health risks in young Indian children
Source: Front Public Health. 2025 May 9;13:1575950. doi: 10.3389/fpubh.2025.1575950 (PMC12098110; doi:10.3389/fpubh.2025.1575950)
Supplement: Supplementary file 1 [file Table_1.docx]

**The role of antioxidant nutrients in mitigating PM_2.5_-related health risks in young Indian children**

Franciosalgeo George^1,2^, Ekta Chaudhary^3^, Sagnik Dey^4,5^, Tinku Thomas^6^, Harshpal Singh Sachdev^7^, Anura Kurpad^8^, *Santu Ghosh^6^

^1^ Centre for Doctoral Studies, Manipal Academy of Higher Education, Manipal, India

^2^ Division of Epidemiology, Biostatistics, and Population Health, St John’s Research Institute, Bangalore, India

^3^ Department of Epidemiology, University of Michigan School of Public Health, Ann Arbor, MI 48109, USA

^4^ Centre for Atmospheric Sciences, Indian Institute of Technology Delhi, New Delhi, India

^5^ Adjunct Faculty, Department of Health, Policy and Management, Korea University, Seoul, South Korea.

^6^ Department of Biostatistics, St John’s Medical College, Bengaluru, India

^7^ Department of Pediatrics and Clinical Epidemiology, Sitaram Bhartia Institute of Science and Research, New Delhi, India

^8^ Department of Physiology, St John’s Medical College, Bengaluru, India

***Correspondence**

Santu Ghosh, Department of Biostatistics, St. John’s Medical College, Sarjapur Road, Bengaluru 560034, India. Tel: +91 80 49466123, Email: [santu.g@stjohns.in](mailto:santu.g@stjohns.in)

**Keywords:** PM_2.5_ Exposure, Acute Respiratory Illness (ARI), Anaemia, Antioxidant Nutrient Intake, Air Pollution, Child Health

**Supplementary Figure 1**: Prevalence of anaemia and ARI among <5y children across different socioeconomic factors (top two panels) and unadjusted and adjusted Odds Ratio of anaemia and ARI for every 10μg/m^3^ increase in ambient PM_2.5_ exposure (bottom left panel), and distribution of life course exposure to ambient PM_2.5_ in <5y children (bottom right panel).


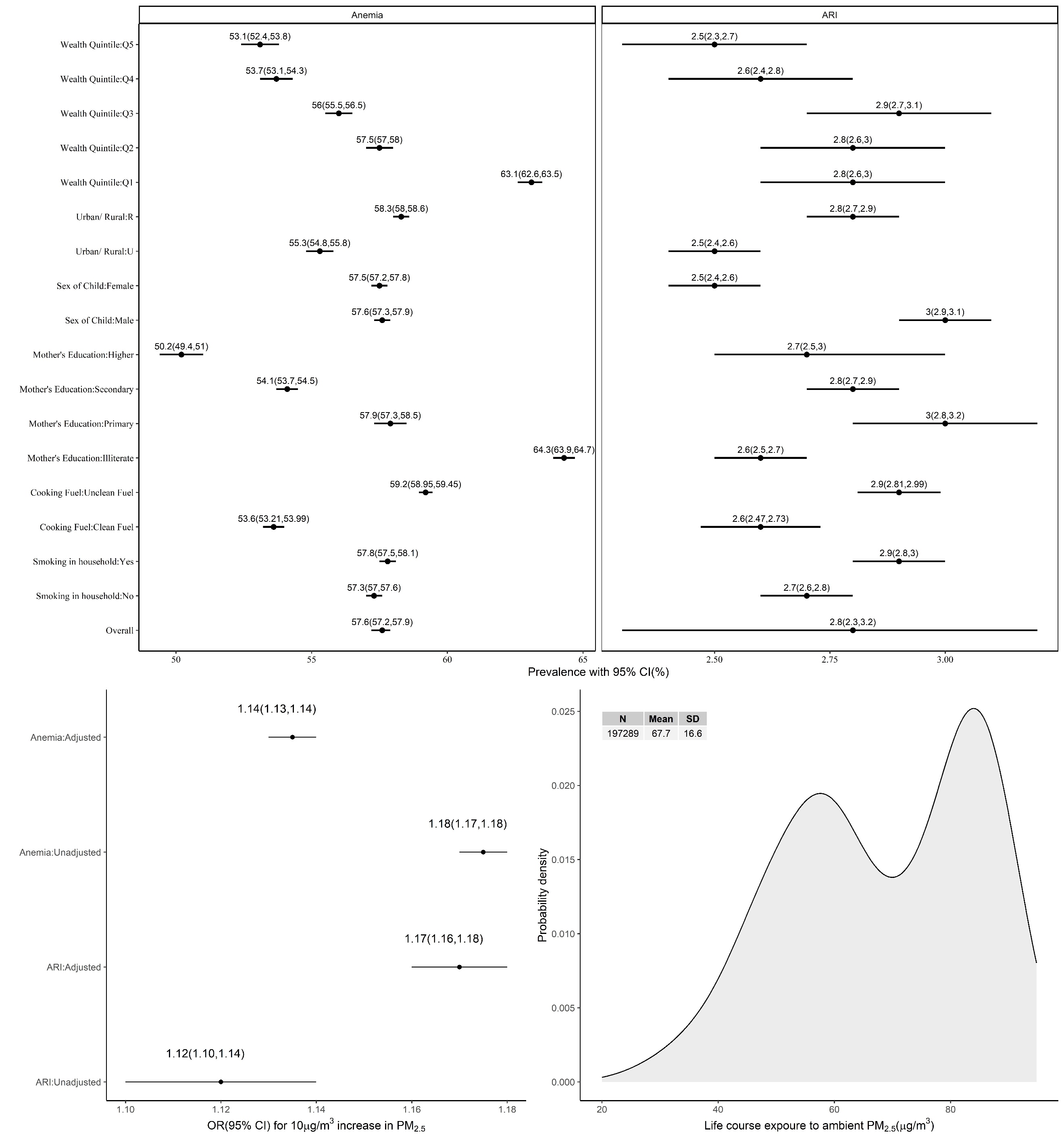


**Supplementary Figure 2**: Distribution of per capita intake of select micronutrients with antioxidant properties (gm: geometric mean; gsd: geometric standard deviation).


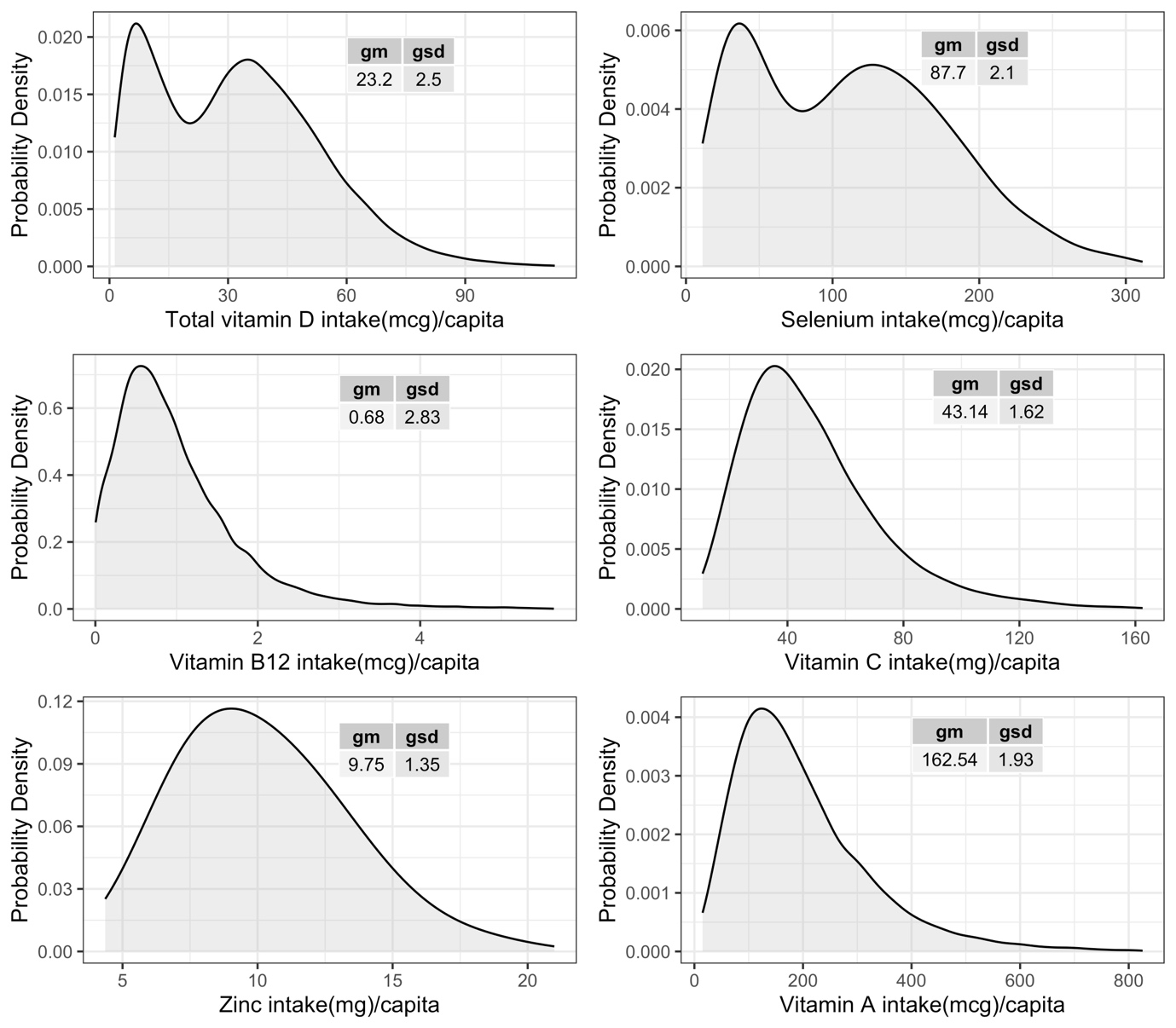


**Supplementary Table 1**: Adjusted^a^ slope of log odds of ARI for every 1μg/m^3^ increase in PM_2.5_ exposure at the selected cut-off^b^ per capita daily intake of select micronutrients and its change at higher levels of intake.

| **Micronutrient** | **Estimate** | **Std. Error** | **P-value** |
| --- | --- | --- | --- |
| ***Vitamin D intake/capita/day*** | | | |
| Slope (PM_2.5_) at intake ≤ 45 μg | 0.0140 | 0.00089 | <0.001 |
| Change in slope (PM_2.5_) at intake > 45 μg | -0.0013 | 0.00039 | <0.001 |
| ***Selenium intake/capita/day*** | | | |
| Slope (PM_2.5_) at intake <160 μg | 0.0140 | 0.00090 | <0.001 |
| Change in slope (PM_2.5_) at intake 160-200 μg | ***-0.0011*** | ***0.00047*** | ***0.021*** |
| Change in slope (PM_2.5_) at intake >200 μg | ***-0.0020*** | ***0.00058*** | ***0.001*** |
| ***Vitamin B_12_ intake/capita/day*** | | | |
| Slope (PM_2.5_) at intake < 0.25 μg | 0.01227 | 0.00101 | <0.001 |
| Change in slope (PM_2.5_) at intake 0.25-0.5 μg | -0.00024 | 0.00069 | 0.734 |
| Change in slope (PM_2.5_) at intake >0.5 μg | 0.00119 | 0.00057 | 0.038 |
| ***Vitamin C intake/capita/day*** | | | |
| Slope (PM_2.5_) at intake ≤ 100 mg | 0.01163 | 0.00090 | <0.001 |
| Change in slope (PM_2.5_) at intake >100 mg | ***-0.00326*** | ***0.00095*** | ***<0.001*** |
| ***Zinc intake/capita/day*** | | | |
| Slope (PM_2.5_) at intake <10 mg | 0.01293 | 0.00095 | <0.001 |
| Change in slope (PM_2.5_) at intake 10-15 mg | 0.00045 | 0.00038 | 0.232 |
| Change in slope (PM_2.5_) at intake >15 mg | -0.00035 | 0.00044 | 0.588 |
| ***Vitamin A intake/capita/day*** | | | |
| Slope (PM_2.5_) at intake < 100 μg | 0.01295 | 0.00093 | <0.001 |
| Change in slope (PM_2.5_) at intake 100-200 μg | 0.00004 | 0.00047 | 0.940 |
| Change in slope (PM_2.5_) at intake >200 μg | 0.00059 | 0.00047 | 0.208 |

a: Adjusted for child age, child's sex, mother's education level, socioeconomic status, type of residence, type of cooking fuel, frequency of smoking within household and household size.

b: See text for description of selection of intake cut-off values

**Supplementary Table 2**: Adjusted^a^ slope of log odds of anaemia for every 1μg/m^3^ increase in PM_2.5_ exposure at the selected cut-off^b^ per capita daily intake of select micronutrients and its change at higher levels of intake.

| **Parameter** | **Estimate** | **Std. Error** | | **p-value** |
| --- | --- | --- | --- | --- |
| ***Vitamin D intake/capita/day*** | | | | |
| Slope (PM_2.5_) at intake ≤ 45 μg | 0.00878 | 0.00032 | <0.001 | |
| Change in slope (PM_2.5_) at intake > 45 μg | ***-0.00370*** | ***0.00022*** | ***<0.001*** | |
| ***Selenium intake/capita/day*** | | | | |
| Slope (PM_2.5_) at intake <160 μg | 0.00952 | 0.00032 | <0.001 | |
| Change in slope (PM_2.5_) at intake 160-200 μg | ***-0.00096*** | ***0.00021*** | ***<0.001*** | |
| Change in slope (PM_2.5_) at intake >200 μg | ***-0.00254*** | ***0.00028*** | ***<0.001*** | |
| ***Vitamin B_12_ intake/capita/day*** | | | | |
| Slope (PM_2.5_) at intake < 0.25 μg | 0.00817 | 0.00044 | <0.001 | |
| Change in slope (PM_2.5_) at intake 0.25-0.5 μg | ***-0.00022*** | ***0.00029*** | ***0.452*** | |
| Change in slope (PM_2.5_) at intake >0.5 μg | ***-0.00086*** | ***0.00035*** | ***0.014*** | |
| ***Vitamin C intake/capita/day*** | | | | |
| Slope (PM_2.5_) at intake ≤ 100 mg | 0.00791 | 0.00033 | <0.001 | |
| Change in slope (PM_2.5_) at intake >100 mg | ***-0.00114*** | ***0.00032*** | ***<0.001*** | |
| ***Zinc intake/capita/day*** | | | | |
| Slope (PM_2.5_) at intake <10 mg | 0.00921 | 0.00035 | <0.001 | |
| Change in slope (PM_2.5_) at intake 10-15 mg | ***-0.00290*** | ***0.00025*** | ***<0.001*** | |
| Change in slope (PM_2.5_) at intake >15 mg | ***-0.00414*** | ***0.00044*** | ***<0.001*** | |
| ***Vitamin A intake/capita/day*** | | | | |
| Slope (PM_2.5_) at intake < 100 μg | 0.00876 | 0.00034 | <0.001 | |
| Change in slope (PM_2.5_) at intake 100-200 μg | ***-0.00167*** | ***0.00018*** | ***<0.002*** | |
| Change in slope (PM_2.5_) at intake >200 μg | ***-0.00251*** | ***0.00020*** | ***<0.003*** | |

a: Adjusted for child age, child's sex, mother's education level, socioeconomic status, type of residence, type of cooking fuel, frequency of smoking within household and household size.

b: See text for description of selection of intake cut-off value
